# Population Assessment of Future Trajectories in Coronary Heart Disease Mortality

**DOI:** 10.1371/journal.pone.0085800

**Published:** 2014-01-21

**Authors:** Rosa Björk Thorolfsdottir, Thor Aspelund, Simon Capewell, Julia Critchley, Vilmundur Gudnason, Karl Andersen

**Affiliations:** 1 Icelandic Heart Association, Kopavogur, Iceland; 2 University of Iceland, Reykjavik, Iceland; 3 Division of Public Health, University of Liverpool, Liverpool, United Kingdom; 4 Department of Population Health Sciences and Education, St George's, University of London, London, United Kingdom; University of Louisville, United States of America

## Abstract

**Background:**

Coronary heart disease (CHD) mortality rates have been decreasing in Iceland since the 1980s, largely reflecting improvements in cardiovascular risk factors. The purpose of this study was to predict future CHD mortality in Iceland based on potential risk factor trends.

**Methods and findings:**

The previously validated IMPACT model was used to predict changes in CHD mortality between 2010 and 2040 among the projected population of Iceland aged 25–74. Calculations were based on combining: i) data on population numbers and projections (Statistics Iceland), ii) population risk factor levels and projections (Refine Reykjavik study), and iii) effectiveness of specific risk factor reductions (published meta-analyses). Projections for three contrasting scenarios were compared: 1) If the historical risk factor trends of past 30 years were to continue, the declining death rates of past decades would level off, reflecting population ageing. 2) If recent trends in risk factors (past 5 years) continue, this would result in a death rate increasing from 49 to 70 per 100,000. This would reflect a recent plateau in previously falling cholesterol levels and recent rapid increases in obesity and diabetes prevalence. 3) Assuming that in 2040 the entire population enjoys optimal risk factor levels observed in low risk cohorts, this would prevent almost all premature CHD deaths before 2040.

**Conclusions:**

The potential increase in CHD deaths with recent trends in risk factor levels is alarming both for Iceland and probably for comparable Western populations. However, our results show considerable room for reducing CHD mortality. Achieving the best case scenario could eradicate premature CHD deaths by 2040. Public health policy interventions based on these predictions may provide a cost effective means of reducing CHD mortality in the future.

## Introduction

In most industrialized countries coronary heart disease (CHD) death rates have declined considerably during the past two to three decades [1]. However, there is evidence that this mortality decline is levelling off, particularly among young adults, for example in USA, Australia, England and Wales [2]–[4]. Furthermore, worldwide, CHD remains the most common cause of death [5]. It is therefore important to focus on the future and try to predict CHD death trends in the next few decades. Over 90% of all cases of myocardial infarction are attributable to a few well known and modifiable risk factors [6]. The main reason for the stalling decline in death rate is adverse trends in some of these established risk factors. In most Western countries, diabetes and obesity have been increasing rapidly, especially among young people [7]–[9]. In some countries adverse trends in other risk factors have also been causing concern, including blood pressure rises among men and persistent smoking among women in the USA [7]. These unfavourable trends might reverse the recent decline in CHD mortality rates.

Ageing of the population increases the lifetime exposure to the risk factors and further threatens the positive mortality trends. This could increase the actual burden of CHD [3]. When predicting future CHD mortality, it is therefore necessary to account for both the ageing population and possible risk factor trends in the coming decades.

In Iceland, there is easy access to reliable data including a large unselected epidemiologic database, the Reykjavik study [10]. In the present study we therefore applied the IMPACT model to the Icelandic population. The IMPACT model has proven its value in linking population risk factor changes to trends in CHD death rates in diverse populations (including England, Ireland, Scotland, Finland, USA, China, New Zealand, Sweden, Poland, the Czech Republic and Iceland), including validation against the actual change in mortality rates observed in these countries [10]–[21]. An analysis comparing estimated mortality falls according to the Icelandic IMPACT model and observed mortality falls in 1981–2006 showed a reasonably good fit [10]. Subsequently, the model has been used to estimate the potential for preventing future CHD deaths by increasing uptake of appropriate treatments or by reductions in specific risk factors in the population [22]–[24].

The purpose of this study was therefore to predict future CHD mortality in Iceland based on potential trends in risk factor levels. We present three distinct scenarios for risk factor changes over the next thirty years and calculate the consequent trends in CHD mortality for each. Predictions of future development in CHD death rates in Iceland may prove relevant for other Western populations, given the fact that cardiovascular risk factors have similar or even identical effects on the pathogenesis of cardiovascular diseases in Iceland as in other Western countries and the very similar impact of specific risk factor trends on cardiovascular death rates [10]–[18], [25]. Furthermore, very similar risk factor impact has been demonstrated in the Eastern European countries of Poland and the Czech Republic, though trends in CHD mortality decline are delayed compared to Western Europe [20]–[21]. Public health policy interventions based on these predictions may provide a cost effective means of reducing CHD mortality in the years to come.

## Methods

### The Reykjavík Study

For the past decades, the Icelandic Heart Association (IHA) has conducted prospective population based cardiovascular surveys, the Reykjavik study and The REFINE Reykjavik study. These are unselected databases representing a large proportion of the general population and they examine conventional cardiovascular risk factors. The design of these studies has been described in detail in previous reports [10].

### Ethical Statement

The REFINE Reykjavik Study was approved by the National Bioethics Committee and all participants gave written informed consent for participation.

### The IMPACT CHD Model

The IMPACT model was designed to explain changes in CHD mortality rates in a population from changes in established risk factors. It calculates how many premature deaths, defined as deaths before the age of 75, would be prevented or postponed because of a certain change in population risk factor level or treatment uptake. The model has been used previously to analyze the decline in CHD mortality rates in Iceland over the years 1981–2006 [10]. In this study, the focus was on risk factor changes exclusively and their specific impact on CHD mortality. Instead of analyzing observed mortality falls in the past we extended the previously calibrated Iceland IMPACT model to the future, in order to predict CHD mortality in different situations. We used three contrasting risk factor development scenarios to calculate deaths prevented or postponed (DPPs) in the Icelandic population aged 25–74 from 2010 to 2020, 2030 and 2040 respectively.

The modeĺs calculations of DPPs from risk factor changes are based on combining the following three sets of variables:

- Expected number of deaths in the final year: Calculated by applying the age specific CHD death rate of 2010 to the expected population in the final year, by sex and 10-year age groups from age 25–74.
- Changes in population levels and prevalence of major cardiovascular risk factors: Smoking: self-reported current smoking, Systolic blood pressure (systolic BP): measured, mmHg, Cholesterol: measured fasting cholesterol, mmol/l, Physical inactivity: self-reported, defined as “not currently exercising regularly”, Body mass index (BMI): measured, kg/m^2^.

Diabetes: defined as self-reported history of diabetes, measured elevated fasting glucose or medication use.

- Effectiveness of specific risk factor changes on CHD mortality based on published meta-analyses. The model employs regression coefficients or relative risk values obtained from multivariate logistic regression analyses. Each coefficient quantifies the independent log linear relationship between the absolute change in a specific cardiovascular risk factor and the consequent change in the number of CHD deaths in the population.

Data sources are summarized in table 1.

**Table 1:** Data sources.

Data	Source
**CHD mortality in 2010**	Statistics Iceland (mean mortality 2007–2009*)
**Population projections 2020, 2030 and 2040**	Statistics Iceland
**Risk factor levels 1981**	Reykjavik study (data from 1979–1983), n = 5391, mean age 53±11
**Risk factor levels 2006**	REFINE Reykjavik study (data from 2005–2007), n = 4161, mean age 60±13
**Risk factor levels 2010**	REFINE Reykjavik study (data from 2009–2011), n = 9552, mean age 52±12

CHD mortality in 2010 was not available.

### Risk Factor Scenarios

Three future risk factor scenarios were designed. They were meant to represent contrasting but feasible possibilities in risk factor development that could be used to calculate possible trends in CHD mortality. The first scenario was based on historical risk factor trends (past 30 years) continuing, the second scenario was based on recent risk factor trends (past five years) continuing, and the third scenario assumed that in 2040 the entire population would enjoy optimal risk factor levels observed in low risk cohorts as defined by Stamler et al and Daviglus et al [26]–[27]. Table 2 provides a comparison of the scenarios. Finally, these three different risk factor scenarios were used to design a fourth scenario, a proposal for obtainable risk factor goals, using the risk factor changes considered to be most effective and realistic.

**Table 2:** Overview of three future risk factor scenarios.

Scenario	Risk factor development
**Historical trends continue:** Assuming risk factor trends ofpast 30 years (1981–2010) continued.	In this 30 year period there was considerable decline in CHD mortality, largely attributable to favourable changes in population risk factor levels (cholesterol, systolic BP, smoking and physical inactivity). However, BMI and diabetes prevalence rose steadily.
**Recent trends continue:** Assuming the recent risk factor trends ofpast five years (2006–2010) continue.	In the past five years there was a relevant plateau in decreasing cholesterol level among both sexes and systolic blood pressure in men stopped falling and has been increasing since 2006. Systolic blood pressure in women has recently declined in a slower rate than previously. Furthermore, the recent increase in BMI and diabetes has accelerated.
**Low risk scenario:** Assuming that the mean risk factor levels ofthe whole population will progressively (35% by 2020, 65% by 2030,and 100% by 2040) reach the optimal levels already reported inlow risk cohorts, as defined by Stamler et al and Davigluset al. [26]–[27]	This includes zero smoking, diabetes and physical inactivity. A systolic BP of 115 mmHg, a total cholesterol of 4.5 mmol/l, and mean BMI of 24 kg/m^2^

### Methodological and Statistical Considerations

Calculations in the IMPACT model were made separately for men and women in 10 year age groups to account for potential differences in effect. Since all the beta coefficients and relative risk values were obtained from multi-variable models they were assumed to be independent. Therefore the DPPs from each risk factor change could be summed. Finally, the total number of CHD-deaths in the final year (2040) was calculated by subtracting the DPPs in that year from the number of deaths expected if 2010 death rates persisted unchanged. The final results were then presented as deaths per 100,000 population.

In this study, calculations included risk factor changes only, not treatments. For example the role of anti-hypertensive and hypercholesterolemia treatments in changes in blood pressure and cholesterol levels were not accounted for. The calculations were based on overall changes in these risk factors, regardless of how they were achieved. It has in fact been demonstrated that changes in total cholesterol levels in Western societies, including Iceland, are not related to statin, but rather dietary factors [28].

Because of the uncertainties surrounding many of the values, multi-way sensitivity analyses were performed using Brigǵs analysis of extremes method.

Further details on the methods and data sources used in this study, as well as examples of the calculations used in the model are provided in Appendix S1.

## Results

If no changes in CHD risk factor levels occurred among the Icelandic population over the coming decades, the same age specific death rates recorded in 2010 would be expected to persist in 2040. This would result in the number of CHD deaths among people aged 25–74 years increasing from approximately 49 to 68 per 100,000 (Table 3), simply reflecting population ageing.

**Table 3:** Population numbers and CHD deaths in Iceland in 2010, and expected population numbers and CHD deaths in 2040 if 2010 rates persist.

	Population in2010	Population in2040	CHD deaths2010	CHD death rate per100.000 2010	CHD deaths in 2040 if2010 rates persisted	CHD death rate per100.000 2040
**Men**						
25–34	23895	25912	0	1.4	0	1.4
35–44	21994	23744	2	10.1	2	10.1
45–54	21860	23975	11	49.4	12	49.4
55–64	16971	22918	22	128.8	30	128.8
65–74	9560	19593	40	419.3	82	419.3
**Total**	**94280**	**116142**	**75**		**126**	
**Women**						
25–34	22666	25012	0	0.0	0	0.0
35–44	21126	23428	0	1.6	0	1.6
45–54	21409	23097	0	1.6	0	1.6
55–64	16353	22505	3	17.5	4	17.5
65–74	10093	19749	13	124.9	25	124.9
**Total**	**91647**	**113791**	**16**		**29**	
**TOTAL**	**185927**	**229933**	**91**	**49**	**156**	**68**

Observed risk factor values in 1981, 2006 and 2010 are summarized in table 4, along with information on the sample sizes and gender and age distribution from the Reykjavik study and REFINE Reykjavik study. Risk factor values in each of the future scenarios are summarized in table 5.

**Table 4:** Data on sample characteristics and risk factor values from the Reykjavik study (1981) and REFINE Reykjavik study (2006 and 2010).

Year	1981		2006		2010	
**n**	5391		4161		4326	
**Mean age (SD)**	53 (11)		60 (13)		52 (12)	
**Males (%)**	47		45		50	
	**Men**	**Women**	**Men**	**Women**	**Men**	**Women**
**Smoking prevalence (%)**	50.8	42.3	23.1	22.1	21.5	19.7
**Mean systolic bp (mmHg)**	128.6	123.4	125.2	116.7	126.1	115.4
**Mean blood cholesterol (mmol/l)**	5.92	6.05	5.16	5.12	5.16	5.18
**Physical inactivity prevalence (%)**	77.8	74.4	54.7	52.9	47.9	43.3
**Mean BMI (kg/m^2^)**	25.4	24.5	27.5	26.5	28.0	26.8
**Diabetes prevalence (%)**	2.0	1.5	4.8	2.4	5.2	3.1

**Table 5:** CHD deaths per 100,000 prevented or added as a result of risk factor changes, under three cardiovascular risk factor scenarios.

Risk factor scenario:			Fewer/additional deaths per 100,000 in 2040				
	Risk factor value		Both sexes			Best estimate	
	Men	Women	Best estimate	*Minimum* *estimate*	*Maximum* *estimate*	Men	Women
**Smoking prevalence** (%)							
In 2010	21.5%	19.7%	–	*–*	*–*	**–**	**–**
Historical trends continue, 2040	8.5%	8.7%	**−7**	*−6*	*−8*	**−6**	**−1**
Recent trends continue, 2040	13.3%	7.9%	**−5**	*−4*	*−6*	**−3**	**−1**
Low risk scenario, 2040	0.0%	0.0%	**−13**	*−11*	*−16*	**−11**	**−2**
**Mean systolic blood pressure** (mmHg)							
In 2010	126.1	115.4	**–**	*–*	*–*	**–**	**–**
Historical trends continue, 2040	123.9	108.7	**−8**	*−5*	*−11*	**−5**	**−3**
Recent trends continue, 2040	130.8	107.1	**3**	*2*	*5*	**7**	**−4**
Low risk scenario, 2040	115.8	112.7	**−24**	*−17*	*−32*	**−20**	**−4**
**Mean blood cholesterol** (mmol/l)							
In 2010	5.16	5.18	**–**	*–*	*–*	**–**	**–**
Historical trends continue, 2040	4.45	4.44	**−12**	*−9*	*−16*	**−10**	**−3**
Recent trends continue, 2040	5.19	5.65	**2**	*1*	*3*	**1**	**2**
Low risk scenario, 2040	4.48	4.48	**−12**	*−8*	*−16*	**−9**	**−3**
**Physical inactivity prevalence** (%)							
In 2010	47.9%	43.3%	**–**	*–*	*–*	**–**	**–**
Historical trends continue, 2040	29.1%	24.9%	**−3**	*−3*	*−4*	**−3**	**−1**
Recent trends continue, 2040	18.2%	9.6%	**−5**	*−4*	*−7*	**−4**	**−1**
Low risk scenario, 2040	0.0%	0.0%	**−9**	*−7*	*−10*	**−7**	**−2**
**Mean BMI** (kg/m^2^)							
In 2010	28.0	26.8	**–**	*–*	*–*	**–**	**–**
Historical trends continue, 2040	31.1	29.5	**4**	*2*	*6*	**3**	**1**
Recent trends continue, 2040	32.7	29.4	**6**	*3*	*9*	**5**	**1**
Low risk scenario, 2040	25.3	23.0	**−4**	*−2*	*−6*	**−3**	**−1**
**Diabetes prevalence** (%)							
In 2010	5.2%	3.1%	**–**	*–*	*–*	**–**	**–**
Historical trends continue, 2040	9.5%	5.3%	**2**	*1*	*2*	**1**	**0**
Recent trends continue, 2040	8.7%	8.6%	**2**	*1*	*2*	**1**	**1**
Low risk scenario, 2040	0.0%	0.0%	**−8**	*−6*	*−9*	**−6**	**−2**
**Totals**							
Historical trends continue, 2040	–	–	**−25**	*−18*	*−32*	**−18**	**−7**
Recent trends continue, 2040	–	–	**3**	*−1*	*7*	**6**	**−3**
Low risk scenario, 2040	–	–	**−71**	*−51*	*−90*	**−56**	**−14**

May not sum to total due to rounding.

### If Historical Trends Continued

The impact of each risk factor change in each scenario is summarized in table 5. If historical trends continued, the beneficial development in mean systolic BP, cholesterol level, and the prevalence of smoking and physical inactivity would result in approximately 30 deaths prevented or postponed (DPPs) per 100,000 in the year 2040. On the other hand, mean BMI would be expected to rise to 30·3 kg/m^2^ and diabetes prevalence would reach 7·5% in 2040, together causing 5 additional deaths per 100,000. The overall outcome would therefore be to decrease the number of deaths in 2040 by 25 per 100,000, a 37% reduction compared to the 68 deaths per 100,000 expected with no risk factor change (Table 5).

### If Recent Trends Continued

Continuing recent risk factor trends of past five years would result in the unfavourable risk factor changes outweighing the benefits of favourable risk factor changes causing a slightly higher death rate compared to no risk factor changes occurring, a total of 3 deaths per 100,000 added (Table 5).

### Low Risk Scenario

Finally, if the ideal scenario was achieved and the entire population could enjoy the optimal risk factor levels observed in low risk individuals, a total of approximately 71 deaths per 100,000 would be prevented or postponed in the year 2040. The greatest contributions would result from lowering systolic blood pressure (34%), smoking prevalence (19%) and cholesterol level (17%). Reaching optimal diabetes prevalence and mean BMI would together reduce deaths by 12 per 100,000 in 2040 (Table 5).

We explored the impact of minimum and maximum model parameters using Brigǵs analysis of extremes method. According to this sensitivity analysis, substantially changing the values influenced the number of deaths prevented or postponed but did not influence the relative contribution of each risk factor change in each scenario (Table 5, columns 5 and 6).

**Figure 1:**
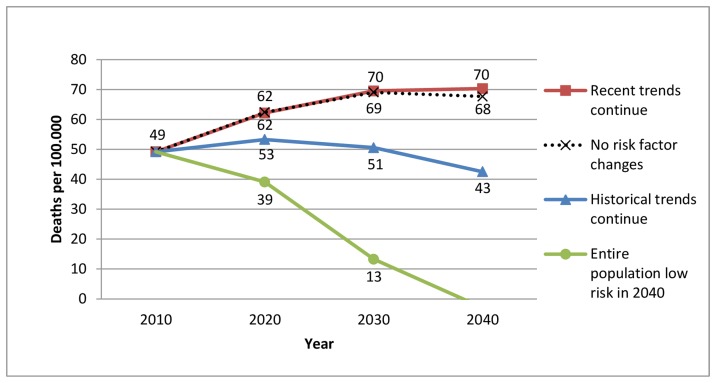
Future CHD mortality rate among 25–74 year old Icelanders in three different risk factor scenarios.

Figure 1 demonstrates how mortality rates would develop from 2010 to 2040 in each risk factor scenario and compares them to the baseline scenario if no risk factor changes were to occur. If the historical risk factor trends were to continue, the declining death rates of past decades would level off. This would be the case despite 25 fewer deaths per 100,000 in 2040 (Table 5) and is primarily explained by the population ageing. Nevertheless, continuing historical trends would result in a lower death rate (43 per 100,000) compared to no risk factor changes (68 per 100,000). Since the trends of past five years are less favourable and result in additional deaths in 2040, continuing those recent trends would result in a death rate increasing from 49 to 70 per 100,000 from 2010 to 2040. In contrast reaching optimal risk factor levels would essentially eliminate all premature preventable CHD deaths before 2040 (Figure 1).

### Obtainable Goals

**Figure 2:**
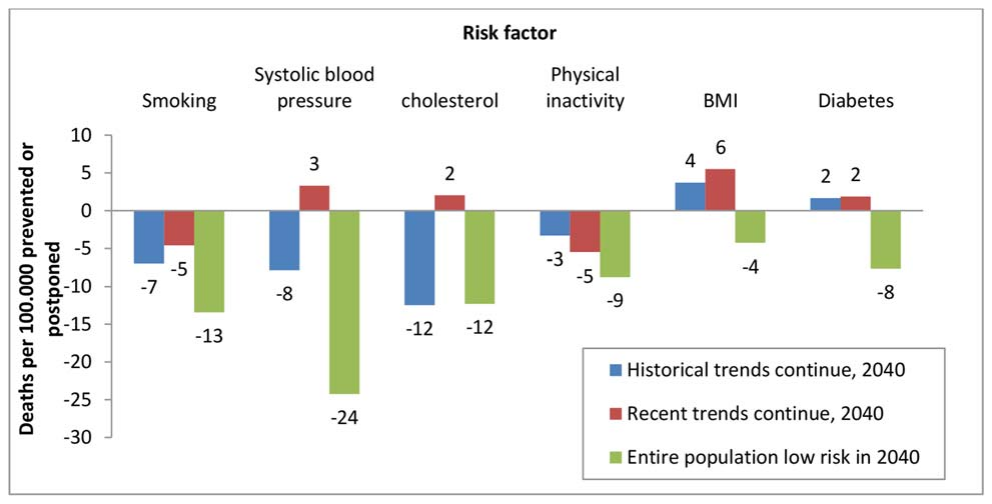
Estimated deaths per 100.000 prevented or added in 2040. Estimated deaths by risk factor change, under three risk factor scenarios.

**Figure 3:**
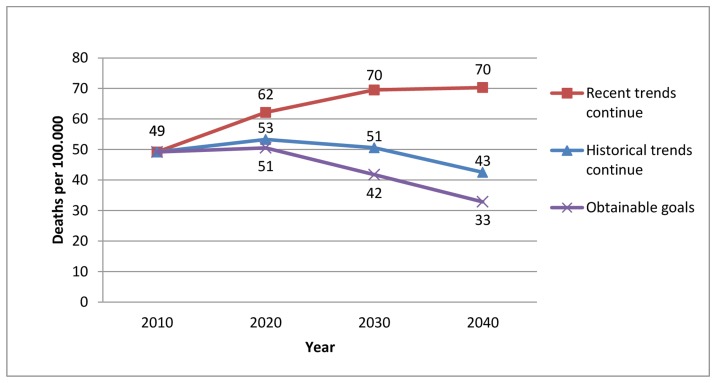
Future CHD mortality rate among 25–74 year old Icelanders. Future CHD mortality in two different risk factor scenarios and a proposal to obtainable goals.

Figure 2 compares the potential effects of changes in each risk factor on the number of deaths in each scenario. A new scenario was made by combining the most effective and realistic risk factor changes in the three different scenarios. These changes are summarized in table 6. The net result of reaching these goals would be to decrease the number of deaths in 2040 by approximately 35 per 100,000 compared to no risk factor changes occurring (Table 6). This would result in death rate decreasing from 49 to 33 per 100,000 from 2010 to 2040 (Figure 3).

**Table 6:** Risk factor levels in 2010 and 2040, and CHD mortality trends from Scenario 4.

Risk factor scenario:	Risk factor value,men	Risk factor value,women	Fewer/add-itional deathsper 100,000 in 2040				Assumed risk factor changes from 2010
			Best estimate	Percentage	Men	Women	
**Smoking prevalence** (%)							Assume low risk scenario (+1%)
2010	21.5	19.7					
2040	**1**	**1**	**−12**	−18%	−10	−2	
**Mean systolic BP** (mmHg)							Men: assume historical trends continue.
2010	126.1	115.4					Women: assume no change from 2010
2040	**123.9**	**115.4**	**−5**	−7%	−5	0	
**Mean blood chol.** (mmol/l)							Low risk scenario
2010	5.16	5.18					
2040	4.48	**4.48**	**−12**	−18%	−9	−3	
**Physical inactivity** (%)							Assume recent trends continue
2010	47.9	43.3					
2040	**18.2**	**9.6**	**−5**	−8%	−4	−1	
**Mean BMI** (kg/m^2^)							Assume no change from 2010
2010	28.0	26.8					
2040	**28.0**	**26.8**	**0**	0%	0	0	
**Diabetes prevalence** (%)							Assume no change from 2010
2010	5.2	3.1					
2040	**5.2**	**3.1**	**0**	0%	0	0	
**Total**			**−35**	**−51%**			

## Discussion

Coronary heart disease death rates in Iceland decreased by 80% during the 25 year period 1981–2006 [29]. This has greatly increased life expectancy and lowered premature deaths. However our results suggest that without significant population lifestyle changes these mortality trends might plateau or even reverse in coming decades. Even if the mostly favourable CHD risk factor trends of the past 30 years were to continue to 2040, the declining death rates of past decades would level off. This surprising result is explained by continuing adverse trends in diabetes and BMI as well as population ageing. Worse still, risk factor trends during the last five years (2006–2010) are less positive than the three previous decades and suggest an alarming trend. In addition to faster increases in diabetes and obesity, cholesterol levels have plateaued and men’s blood pressure is actually rising. If these recent trends continue to 2040 we are facing a public health disaster, where CHD mortality will increase and the gains of past decades will be lost. However there is room for considerable improvement as shown by the more optimistic scenario assuming that in 2040 the entire population enjoys risk factor levels currently observed in low risk individuals.

### Mortality and Risk Factor Development

The majority (74%) of the reduced mortality between 1981 and 2006 was explained by improvement in risk factors in the community. A decrease in blood cholesterol, explained approximately 32% of the reduction, and cigarette smoking and systolic BP, each explained some 22% of the reduction [10]. Adverse Icelandic trends during the last five years cause great concern. Unopposed, the recent plateau in cholesterol levels will cause a 3% increase in deaths in 2040 and increasing blood pressure among men may increase mortality by 10%. These adverse trends may reflect a shift to a more unhealthy diet, following the financial crisis in 2008. Furthermore, if recent rising trends in diabetes and obesity continue, they will result in an 11% increase in deaths in 2040, echoing similar concerns in other Western countries, including the USA [7]. It has been predicted by Olshansky and colleagues that life expectancy in the United States could level off or even decline within the first half of the 21st century as a result of the substantial rise in the prevalence of obesity and it’s life-shortening complications, such as diabetes [30]. Because of the transferability of the results, the possibly disastrous future development in CHD death rates is alarming both for Iceland and for the majority of Western populations.

### Preventing Premature Deaths in the Future

Despite a pessimistic view of the future, our results also show considerable room for improvement. The scenarios are not equally realistic. For example, according to the low risk scenario, men‘s mean systolic blood pressure will reach 115·8 mmHg in 2040, perhaps an unrealistic and not very effective goal. In fact assuming that the entire population obtains a low risk profile is a highly unrealistic scenario especially in light of recent concerning risk factor trends in Iceland. Furthermore, the total outcome of this scenario would be to eliminate all premature CHD deaths before 2040. This would be the result of risk factor changes only, despite the fact that 25% of the mortality decline in Iceland the past (1981–2006) has been attributed to treatment effects [10]. However, it can be argued that treatment uptake is dependent on risk factor levels and disease prevalence so that lowering risk factor values would decrease the overall importance of treatment interventions. Furthermore, the purpose of presenting the low risk scenario was not to put forward a realistic future vision where the low risk factor goals would be achieved in all age and sex groups but rather to use the calculations to demonstrate which risk factor changes are most effective and realistic. They where then used to construct a fourth scenario based on the most efficient and realistic risk factor changes from the other scenarios. This is in line with the targets put forward by the World Health Organization for prevention and control of non-communicable diseases [31]. The dramatic net result of reaching these obtainable goals would be halving the expected death rates in 2040, from 68 per 100.000 to 33 per 100.000. This eloquently demonstrates that further reductions in CHD mortality are possible, despite the population ageing, even when mortality rates approach zero and further mortality decreases become harder to achieve.

In order to achieve these new goals we need to commit to a multi sectored effort involving various institutions and governmental interventions. In general, the most effective approach to cardiovascular disease prevention is risk factor reduction in apparently healthy people (population based prevention) rather than in individuals at high risk of future CHD events [32]. The focus must therefore be on tobacco control, healthy diet and exercise. It is important to start early and provide children with healthy school meals and physical education. In order to secure continuing drops in smoking rates, it is important to further tighten the legislation and invest in preventive measures, including plain packaging and licensing of retail outlets. Food products must be priced according to their wholesomeness and healthy food should be made more visible and accessible. A recently approved regulation that limits the amount of *trans* fats in foods to two grams per 100 grams will hopefully have a positive effect on cholesterol values in the future. Similar laws have existed in Denmark for several years with very good results [33].

### Strengths and Limitations of this Study

Modelling studies have a number of strengths. They offer the potential to further our understanding of coronary heart disease epidemiology and can transparently consider and simultaneously integrate large amounts of data from many sources. Nevertheless they can be considered a crude simplification of a complex reality and there are many assumptions involved in this approach. One for example is to apply results from primary research studies to different populations and assuming that concomitant risk factor reductions are independent. Explicit assumptions can however be tested by sensitivity analysis as is done here [34]. Furthermore, the long tradition of high quality national population surveys and registries in Iceland reduces the potential problem of needing to make assumptions about dubious data. This study focuses on future CHD mortality, thus implying more uncertainties compared to studying the past. As an example, here we only consider the effects of risk factor changes and assume that the effect of treatment will remain unchanged. We therefore assume that even future advances in treatment would not substantially reduce the importance of risk factor improvements and the subsequent gains.

Despite aforementioned concerns over increased burden of CHD due to population ageing this study focuses on premature deaths. This was done for several reasons. The original IMPACT model includes only those adults aged 25–74 years old because of very limited data in older groups [10]. Furthermore, the modelling of deaths prevented or postponed becomes increasingly complicated and unreliable with extremes in age due to comorbidity and other complicating factors. However, changes in age composition of people aged 25–74 is accounted for. Also, it can be argued that risk factor changes among those under 75 years old will influence the health and death rate among this group as it grows older.

## Conclusions

If the mostly favourable CHD risk factor trends of the past 30 years in Iceland were to continue, the declining death rates of past decades would level off, reflecting population ageing and continuing rises in diabetes and BMI. The potential future increase in mortality with recent adverse trends in risk factor levels is alarming and potentially relevant to other Western populations. However, our other scenarios offer considerable room for reducing future CHD mortality, especially by evidence-based population-wide preventive interventions. Achieving the best case scenario could essentially eradicate premature CHD deaths by 2040.

## Supporting Information

Appendix S1 **Supplementary appendix for the Icelandic IMPACT model.** Population assessment of future trajectories in coronary heart disease mortality.
(DOCX)
