# Supplementary material for: Population Assessment of Future Trajectories in Coronary Heart Disease Mortality
Source: PLoS One. 2014 Jan 21;9(1):e85800. doi: 10.1371/journal.pone.0085800 (PMC3897505; doi:10.1371/journal.pone.0085800)
Supplement: Appendix S1 — Supplementary appendix for the Icelandic IMPACT model. Population assessment of future trajectories in coronary heart disease mortality. (DOCX) [file pone.0085800.s001.docx]

| 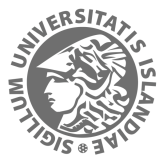  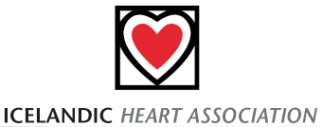[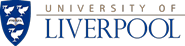](http://www.liv.ac.uk/)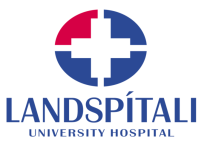  **IMPACT, A VALIDATED, comprehensive CORONARY HEART DISEASE MODEL**  **SUPPLEMENTARY APPENDIX**  **for the Icelandic MODEL:**  Population assessment of future trajectories in coronary heart disease mortality  **Rosa Bjork Thorolfsdottir, Thor Aspelund, Simon Capewell, Julia Critchley, Vilmundur Gudnason, Karl Andersen**  **2013** |
| --- |

**SUPPLEMENTARY APPENDIX FOR THE IMPACT MODEL**

|  |  | |
| --- | --- | --- |
| **Contents** |  | **Page** |
| Table 1. | The Icelandic IMPACT Model: Introduction, detailed methodology and examples of future deaths prevented or postponed (DPP) calculations | 3 |
| Table 2.  Table 3a  Table 3b  Table 4a  Table 4b  Table 5a  Table 5b | Main data sources for the parameters used in the Icelandic IMPACT Model **Men´s risk factor values by age group: Historical trends continue**  **Women´s risk factor values by age group: Historical trends continue**  **Men´s risk factor values by age group: Recent trends continue**  **Women´s risk factor values by age group: Recent trends continue**  **Men´s risk factor values by age group: Low risk scenario**  **Women´s risk factor values by age group: Low risk scenario** | 13 |
| Table 6. | Specific beta coefficients for major risk factors:Data sources, values and comments. |  |
| Table 7. | **Relative risk values for CHD mortality: smoking, diabetes and physical inactivity** (Best, minimum and maximum estimates from InterHeart) |  |
| Table 8. | Icelandic IMPACT Model Risk Factor Methodology: Rationale for choice of regression or PARF approaches for specific risk factors |  |
|  |  |  |
|  |  |  |
|  |  |  |
|  |  |  |

Table 1. The ICELANDIC IMPACT MODEL: INTRODUCTION, DETAILED METHODOLOGY AND EXAMPLE OF FUTURE DEATHS PRVENTED OR POSTPONED (DPP) CALCULATIONS

The tables included in this supplementary appendix document provide details about the methods that were used in creating the Icelandic IMPACT model for calculating future coronary heart disease (CHD) deaths. Earlier versions of the IMPACT mortality model have been previously applied to data from Europe, New Zealand, USA and China in order to analyse the effects of changes in risk factors and treatments on observed mortality falls in the past.[^1-9^](#_ENREF_1) This version of the model focuses on the risk factor changes only and is extended to the future in order to predict CHD mortality in different situations. We used three contrasting risk factor development scenarios to calculate deaths prevented or postponed (DPPs) in the Icelandic population aged 25-74 from 2010 to 2020, 2030 and 2040 respectively. This cell-based mortality model, developed in Microsoft Excel, has been described in detail online and elsewhere.[^1^](#_ENREF_1)^,^[^2^](#_ENREF_2)^,^[^10^](#_ENREF_10)

**Predicted changes in mortality rates from CHD, in Iceland from 2010-2040**

Data sources used in predicting the changes in mortality rates from 2010 to 2040 among Icelandic adults aged 25-74 years are shown in Table 2. These include CHD mortality rates in 2010, population risk factor levels in 2010 and in the past (1981 and 2006 - used to design future risk factor scenarios) and population numbers and projections. Mortality rates from CHD were calculated using the underlying cause of death: International Classification of Diseases (ICD)-10 codes I20-I25. Both unadjusted and age-adjusted mortality rates were calculated. Age-standardization was done using the direct method.

**Expected number of deaths from CHD and deaths prevented or postponed (DPPs)**

The expected number of deaths from CHD in the final year (2020, 2030 or 2040) was calculated by multiplying the age-specific mortality rates from CHD in 2010 by the population projections for the final year in that age-stratum. Summing over all age strata then yielded the expected numbers of deaths from CHD. The future risk factor scenarios were then applied to the model in order to calculate deaths prevented or postponed in the final year. The number of DPPs in the final year could then be subtracted from the number of deaths expected giving the total number of deaths in that year according to each scenario. Finally, all numbers of deaths were presented as deaths per 100.000.

**Risk factor scenarios**

The Impact model estimates the number of coronary heart disease DPPs related to changes in cardiovascular risk factor levels in the population. The Icelandic IMPACT model includes total cholesterol, smoking, systolic blood pressure, body mass index (BMI), diabetes, and physical inactivity. In this study, three contrasting risk factor scenarios were designed in order to predict changes in CHD mortality between 2010 and 2040 among the projected population of Iceland aged 25-74. They each represented a different risk factor development: 1) if historical risk factor trends of past 30 years were to continue, 2) if recent risk factor trends of past five years were to continue and 3) assuming **that in 2040 the entire population enjoys risk factor levels observed in low risk individuals.**  Explanations of each scenario and the methods used to design them are as follows:

Historical trends continue:

This scenario was based on projecting the historical trends in risk factor levels between 1981 and 2010 to the future. In this 30 year period there was considerable decline in CHD mortality, largely attributable to favorable changes in population risk factor levels. Mean cholesterol levels and systolic blood pressure dropped, as well as the prevalence of smoking and physical inactivity. On the other hand, BMI and diabetes prevalence have been rising steadily. Data for 1981 had already been used to analyse the decline in CHD deaths in Iceland between 1981 and 2006, and were therefore easily accessible. 2010, on the other hand, was the chosen starting point for this research.

For each risk factor, the average relative change per year in the period from 1981 to 2010 was calculated for both sexes in ten year age groups. For each sex we then computed the weighted average of the relative change per year over the age groups and applied that change to each age group between 2010 and 2040. This results in an exponential increase or decrease. For example, between 1981 and 2010 the weighted average relative change in systolic blood pressure among men was a decrease by 0.08% per year. When applied to men aged 65-74 years, the mean levels of blood pressure were predicted to change from 133.8 mmHg in 2010 to 130.7 mmHg in 2040. The computations were 133.8 * 0.9992^30 = 130.7. This calculation was then repeated for men and women in each age group. However, there was one exception from using this method, in the case of diabetes. Projecting relative changes in the prevalence of diabetes to the future would have given absurd values (for example many thousand per cent of the youngest women having diabetes in 2040). Therefore the absolute change in diabetes prevalence was projected to the future, instead of using the relative change, giving a much more realistic result.

When projecting past trends to the future it has to be kept in mind that certain risk factor levels are unrealistic or even harmful if they become too low or too high. For example, mean systolic blood pressure reaching 80 mmHg in 2040 would neither be realistic nor would it be useful in lowering CHD mortality. The IMPACT model makes no distinction between a 5 mmHg lowering of systolic blood pressure in the range of 140 to 135 or 85 to 80, calculated DPP´s would be the same. Therefore it is necessary to make sure that risk factor levels are realistic before they are used to calculate DPP´s. That was done by comparing the most extreme future values to the highest or lowest values observed in existing populations.

Recent trends continue: This scenario projects the recent risk factor trends between 2006 and 2010 to the future. The recent trends of past five years differ from the historical trends of past 30 years in a few ways. The prevalence of smoking and physical inactivity dropped in similar levels, but there was a plateau in decreasing cholesterol level among both sexes and systolic blood pressure in men stopped declining and has been increasing since 2006. Systolic blood pressure in women declined in a slower rate than in the whole 30 year period and the increase in BMI and diabetes was faster. The same methods were used to design this scenario as the historical trends scenario.

Low risk scenario: This optimistic scenario assumes that step by step the mean risk factor levels of the whole population will reach the optimal levels already reported in low risk cohorts, as defined by Stamler et al and Daviglus et al.[^11^](#_ENREF_11)^,^[^12^](#_ENREF_12) According to this scenario, in 2020 35% of the population will enjoy the mean risk factor levels of low risk individuals and in 2030 it will be 65%. In the end, the whole population will have the low risk mean.

Risk factor values of both sexes and all age groups in each scenario are summarized in tables 3 (a,b) – 5 (a,b) . Figure 1 demonstrates past risk factor trends and predicted risk factor development up to 2040 according to the three different risk factor scenarios.

**Calculating DPPs based on risk factor changes**

Two approaches to calculating DPPs from changes in risk factors were used.

In the **regression approach**—used for systolic blood pressure, total cholesterol, and body mass index—the number of deaths from CHD occurring in 2010 (the base year) were multiplied by the absolute change in risk factor prevalence, and by a regression coefficient quantifying the change in CHD mortality that would result from the change in risk factor level. Natural logarithms were used, as is conventional, in order to best describe the log-linear relationship between changes in risk factor levels and mortality.

**Example 1: estimation of DPPs from risk factor change using regression method:**

***Mortality fall due to reduction in systolic blood pressure in women aged 65-74 if historical trends in risk factor are to continue from 2010 to 2040.***

For example, if 2010 death rates persisted, 25 CHD deaths are expected in 2040 among 19749 women aged 65-74. If historical trends are to continue, mean systolic blood pressure in this group will decrease by 8,71 mmHg in the period (from 128,540 in 2010 to 119,829 in 2040). The largest meta-analysis reports an estimated age- and sex-specific reduction in mortality of 52 per cent for every 20 mmHg reduction in systolic blood pressure, generating a logarithmic coefficient of -0.032.[^13^](#_ENREF_13)

The number of deaths prevented or postponed in 2040 as a result of this change was therefore estimated as:

= (1-(EXP(coefficient*change))*deaths expected in 2040 if 2010 mortality rates persist.

= (1-EXP(-0.032*8.71 ))* 25 = 6

This calculation was then repeated

a) for men and women in each age group, and

b) using maximum and minimum values in each group, to generate a sensitivity analysis.

Data sources for the number of CHD deaths are shown in Table 2, sources for the population means of risk factors are shown in Table 2, and sources for the coefficients used in these analyses are listed in Table 6.

**Example 2: estimation of DPPs from risk factor change using PARF method.**

***Smoking in men aged 65-74 years, historical trends continue from 2010 to 2040.***

The **population-attributable risk factor (PARF) approach** was used for smoking, diabetes, and physical activity. PARF was calculated conventionally as

**(P x (RR-1)) / (1+P x (RR-1))**

where P is the prevalence of the risk factor and RR is the relative risk for CHD mortality associated with that risk factor. DPPs were then estimated as the CHD deaths in 2040 if 2010 rates persisted multiplied by the difference in the PARF for 2010 and 2040.

For example, the prevalence of smoking among men aged 65-74 years was 13.5% in 2010 and if historical trends in risk factors continue, will be 5.5% in 2040. Assuming a Relative Risk of 2.52[^14^](#_ENREF_14), the PARF was 0.170 in 2010 and 0.078 in 2040. The number of deaths prevented or postponed attributable to the decrease in smoking prevalence from 2010 to 2040 would therefore be the CHD deaths expected in 2040 if 2010 rates persist, (82) * (0.170-0.078 ) = 8 DPPs

This calculation was then repeated

a) for men and women in each age group,

b) using maximum and minimum values in each group, to generate a sensitivity analysis

Data sources for the prevalence of risk factors and for the number of CHD deaths are shown in Table 2. Sources for the relative risks used in these PARF analyses are listed in Table 7 All come from the InterHeart study,[^14^](#_ENREF_14) the largest international study to provide independent RR values, adjusted for other major risk factors. The rationale for choosing the regression or PARF approaches for specific risk factors in the Icelandic IMPACT Model is detailed in Table 8.

**Other Methodological Considerations**

**Sensitivity Analyses**

Because of the uncertainties surrounding many of the values, multi-way sensitivity analyses were performed using Brigg’s analysis of extremes method.[^15^](#_ENREF_15)

Minimum and maximum mortality reductions were generated for effectiveness of risk factor change, using 95% confidence intervals for β coefficients obtained from the most recent meta-analyses or large randomised controlled trials. Minimum and maximum plausible values for the remaining key parameters, relative risk, change in risk factor and CHD death numbers in 2010, the base year, reflected the quality of the available data. Current default values in the IMPACT Model are + 20% for relative risk and change in risk factor.

An analysis of extremes was therefore performed whereby the maximum and minimum feasible values were fed in to the model. By multiplying through, the resulting product then generated maximum and minimum estimates for deaths prevented or postponed (Table below).

**Example: sensitivity analysis for systolic blood pressure levels among 65-74 year old men, historical trends in risk factors continue from 2010 to 2040.**

An example of calculating lower and upper bound estimates for DPPs for change in systolic blood pressure among men, 65-74 year old if historical trends in risk factors continue from 2010 to 2040. 95% confidence intervals for the β coefficient from the meta-analysis were used for relative mortality reduction; lower and upper bound estimates for the other parameters were calculated as minus or plus 20% [except for CHD deaths expected in 2040 if 2010 death rates would continue]. Multiplying all the lower-bound estimates yielded the minimum [lower bound] estimate and multiplying the upper-bound estimates yielded the maximum [upper bound] estimate.

|  | **CHD deaths in 2040 if 2010 rates persisted** | **Beta- coefficient** | **Risk factor change** | **Deaths prevented**  **or postponed** |
| --- | --- | --- | --- | --- |
|  | **A** | **B** | **C** | **(1-(EXP(C*B)))*A** |
| Best Estimate | 82 | 0.027 | 3.11 | 6.6 |
| Minimum estimate | 82 | 0.022^a^ | 2.49 | 4.3 |
| Maximum estimate | 82 | 0.033^a^ | 3.73 | 9.3 |

^a^lower and Upper 95% CI from Prospective studies collaborative meta-analysis, Lancet 2002.[^13^](#_ENREF_13) See Table 6.

This approach may be described as a “robust” approach for two reasons.

a) maximum and minimum values for each variable were deliberately forced to provide a wider range rather than a narrower one, e.g. risk factor change +20% rather than say, +10%.

b) the resulting product, for instance the minimum estimate, was generated by assuming that the lowest feasible values all occurred at the same time, a most unlikely situation.

| Table 2. Main Data Sources for the Parameters Used in the Icelandic IMPACT Model   \| **Data** \| **Source** \| \| --- \| --- \| \|  \|  \| \| **CHD mortality in 2010** \| Statistics Iceland (mean mortality 2007-2009*) \| \| **Population numbers (2010) and projections (2020, 2030 and 2040)** \| Statistics Iceland \| \| **Risk factor levels 1981, 2006 and 2010** \| IHA (data from 1979-1983, 2005-2006 and 2009-2011 respectively) \| \|  \|  \|   *CHD mortality 2010 was not available  IHA Icelandic Heart Association and LSH National University Hospital in Reykjavík. | | |  |
| --- | --- | --- | --- | --- | --- | --- | --- | --- | --- | --- | --- | --- | --- | --- | --- |
|  |  |  |  |
|  |  |  |  |
|  |  |  |  |
|  |  |  |  |
|  | |  |  |
|  |  |  |  |
|  |  |  |  |
|  |  |  |  |
|  |  |  |  |
|  |  |  |  |
|  |  |  |  |
|  | |  |  |
|  |  |  |  |
|  |  |  |  |
|  |  |  |  |
|  |  |  |  |
|  |  |  |  |
|  |  |  |  |
|  |  |  |  |
|  |  |  |  |
|  |  |  |  |
|  |  |  |  |

**Table 3a. Men´s risk factor values by age group: Historical trends continue**

| **Age** | **1981** | **2010** | **Relative change per year** | **Weighted mean** | **2020** | **2030** | **2040** |
| --- | --- | --- | --- | --- | --- | --- | --- |
| SBP |  |  |  |  |  |  |  |
| 25-34 | 120.9 | 123.5 | 1.00074 | 0.99922 | 122.6 | 121.6 | 120.7 |
| 35-44 | 122.4 | 124.4 | 1.00058 | 0.99922 | 123.4 | 122.5 | 121.5 |
| 45-54 | 131.4 | 124.5 | 0.99812 | 0.99922 | 123.5 | 122.5 | 121.6 |
| 55-64 | 136.8 | 129.5 | 0.99811 | 0.99922 | 128.5 | 127.5 | 126.5 |
| 65-74 | 147.1 | 133.8 | 0.99673 | 0.99922 | 132.8 | 131.7 | 130.7 |
| Smoking |  |  |  |  |  |  |  |
| 25-34 | 55.7% | 25.0% | 0.97273 | 0.97080 | 18.6% | 13.8% | 10.3% |
| 35-44 | 55.2% | 21.6% | 0.96812 | 0.97080 | 16.0% | 11.9% | 8.9% |
| 45-54 | 48.7% | 23.2% | 0.97465 | 0.97080 | 17.2% | 12.8% | 9.5% |
| 55-64 | 46.3% | 18.9% | 0.96963 | 0.97080 | 14.1% | 10.5% | 7.8% |
| 65-74 | 37.4% | 13.5% | 0.96537 | 0.97080 | 10.0% | 7.4% | 5.5% |
| PI |  |  |  |  |  |  |  |
| 25-34 | 70.6% | 43.5% | 0.98345 | 0.98297 | 36.6% | 30.8% | 26.0% |
| 35-44 | 77.3% | 41.2% | 0.97854 | 0.98297 | 34.7% | 29.2% | 24.6% |
| 45-54 | 82.8% | 51.9% | 0.98400 | 0.98297 | 43.7% | 36.8% | 31.0% |
| 55-64 | 82.9% | 55.0% | 0.98598 | 0.98297 | 46.3% | 39.0% | 32.9% |
| 65-74 | 84.4% | 53.2% | 0.98423 | 0.98297 | 44.8% | 37.8% | 31.8% |
| Chol. |  |  |  |  |  |  |  |
| 25-34 | 5.56 | 4.80 | 0.99494 | 0.99505 | 4.57 | 4.35 | 4.14 |
| 35-44 | 5.91 | 5.23 | 0.99579 | 0.99505 | 4.98 | 4.74 | 4.51 |
| 45-54 | 6.14 | 5.45 | 0.99590 | 0.99505 | 5.19 | 4.94 | 4.70 |
| 55-64 | 6.29 | 5.29 | 0.99405 | 0.99505 | 5.03 | 4.79 | 4.56 |
| 65-74 | 6.11 | 5.05 | 0.99345 | 0.99505 | 4.81 | 4.57 | 4.35 |
| BMI |  |  |  |  |  |  |  |
| 25-34 | 24.6 | 27.2 | 1.00347 | 1.00329 | 28.1 | 29.0 | 30.0 |
| 35-44 | 25.3 | 27.8 | 1.00324 | 1.00329 | 28.7 | 29.7 | 30.7 |
| 45-54 | 25.9 | 28.1 | 1.00284 | 1.00329 | 29.0 | 30.0 | 31.0 |
| 55-64 | 26.1 | 28.8 | 1.00332 | 1.00329 | 29.7 | 30.7 | 31.8 |
| 65-74 | 26.1 | 29.3 | 1.00395 | 1.00329 | 30.2 | 31.3 | 32.3 |
| Diabetes |  |  | Absolute change per year |  |  |  |  |
| 25-34 | 0.2% | 0.0% | -0.05% | 1.08% | 1.1% | 2.2% | 3.2% |
| 35-44 | 0.5% | 2.8% | 0.81% | 1.08% | 3.9% | 5.0% | 6.1% |
| 45-54 | 1.5% | 4.6% | 1.06% | 1.08% | 5.7% | 6.7% | 7.8% |
| 55-64 | 4.0% | 11.2% | 2.47% | 1.08% | 12.3% | 13.3% | 14.4% |
| 65-74 | 8.7% | 14.8% | 2.09% | 1.08% | 15.8% | 16.9% | 18.0% |

**Table 3b. Women´s risk factor values by age group: Historical trends continue**

| **Age** | **1981** | **2010** | **Relative change per year** | **Weighted mean** | **2020** | **2030** | **2040** |
| --- | --- | --- | --- | --- | --- | --- | --- |
| SBP |  |  |  |  |  |  |  |
| 25-34 | 112.8 | 109.6 | 0.99901 | 0.99766 | 107.1 | 104.6 | 102.2 |
| 35-44 | 115.3 | 111.0 | 0.99869 | 0.99766 | 108.4 | 105.9 | 103.5 |
| 45-54 | 125.4 | 115.5 | 0.99716 | 0.99766 | 112.8 | 110.2 | 107.7 |
| 55-64 | 134.4 | 120.9 | 0.99637 | 0.99766 | 118.1 | 115.4 | 112.7 |
| 65-74 | 145.9 | 128.5 | 0.99565 | 0.99766 | 125.6 | 122.7 | 119.8 |
| Smoking |  |  |  |  |  |  |  |
| 25-34 | 48.9% | 23.9% | 0.97559 | 0.97396 | 18.3% | 14.1% | 10.8% |
| 35-44 | 45.0% | 16.2% | 0.96539 | 0.97396 | 12.4% | 9.6% | 7.3% |
| 45-54 | 40.6% | 21.1% | 0.97769 | 0.97396 | 16.2% | 12.4% | 9.6% |
| 55-64 | 36.2% | 20.2% | 0.98006 | 0.97396 | 15.5% | 11.9% | 9.2% |
| 65-74 | 32.3% | 13.6% | 0.97046 | 0.97396 | 10.4% | 8.0% | 6.1% |
| PI |  |  |  |  |  |  |  |
| 25-34 | 63.1% | 41.6% | 0.98575 | 0.98122 | 34.4% | 28.5% | 23.6% |
| 35-44 | 72.2% | 37.5% | 0.97768 | 0.98122 | 31.0% | 25.7% | 21.2% |
| 45-54 | 79.7% | 45.5% | 0.98083 | 0.98122 | 37.6% | 31.1% | 25.7% |
| 55-64 | 82.3% | 45.2% | 0.97952 | 0.98122 | 37.4% | 30.9% | 25.6% |
| 65-74 | 87.7% | 51.8% | 0.98203 | 0.98122 | 42.9% | 35.5% | 29.3% |
| Chol. |  |  |  |  |  |  |  |
| 25-34 | 5.36 | 4.54 | 0.99429 | 0.99445 | 4.29 | 4.06 | 3.84 |
| 35-44 | 5.74 | 4.83 | 0.99407 | 0.99445 | 4.57 | 4.32 | 4.09 |
| 45-54 | 6.26 | 5.49 | 0.99548 | 0.99445 | 5.19 | 4.91 | 4.65 |
| 55-64 | 6.82 | 5.76 | 0.99419 | 0.99445 | 5.45 | 5.15 | 4.87 |
| 65-74 | 6.90 | 5.76 | 0.99379 | 0.99445 | 5.45 | 5.15 | 4.87 |
| BMI |  |  |  |  |  |  |  |
| 25-34 | 23.1 | 25.7 | 1.00372 | 1.00299 | 26.5 | 27.3 | 28.1 |
| 35-44 | 24.1 | 26.7 | 1.00357 | 1.00299 | 27.5 | 28.4 | 29.2 |
| 45-54 | 25.1 | 27.1 | 1.00259 | 1.00299 | 27.9 | 28.7 | 29.6 |
| 55-64 | 25.8 | 27.3 | 1.00194 | 1.00299 | 28.2 | 29.0 | 29.9 |
| 65-74 | 26.2 | 28.3 | 1.00266 | 1.00299 | 29.2 | 30.0 | 31.0 |
| Diabetes |  |  | Absolute change per year |  |  |  |  |
| 25-34 | 0.0% | 1.0% | 0.00333 | 0.00551 | 1.5% | 2.1% | 2.6% |
| 35-44 | 0.0% | 1.3% | 0.00442 | 0.00551 | 1.9% | 2.4% | 3.0% |
| 45-54 | 0.5% | 2.8% | 0.00809 | 0.00551 | 3.4% | 3.9% | 4.5% |
| 55-64 | 3.3% | 4.1% | 0.00270 | 0.00551 | 4.6% | 5.2% | 5.7% |
| 65-74 | 6.9% | 10.3% | 0.01179 | 0.00551 | 10.9% | 11.4% | 12.0% |

**Table 4a. Men´s risk factor values by age group: Recent trends continue**

| **Age** | **2006** | **2010** | **Relative change per year** | **Weighted mean** | **2020** | **2030** | **2040** |
| --- | --- | --- | --- | --- | --- | --- | --- |
| SBP |  |  |  |  |  |  |  |
| 25-34 | 121.5 | 123.5 | 1.00421 | 1.00103 | 124.8 | 126.1 | 127.4 |
| 35-44 | 122.5 | 124.4 | 1.00396 | 1.00103 | 125.7 | 127.0 | 128.3 |
| 45-54 | 123.8 | 124.5 | 1.00135 | 1.00103 | 125.8 | 127.1 | 128.4 |
| 55-64 | 129.5 | 129.5 | 0.99996 | 1.00103 | 130.8 | 132.2 | 133.5 |
| 65-74 | 140.7 | 133.8 | 0.98753 | 1.00103 | 135.2 | 136.6 | 138.0 |
| Smoking |  |  |  |  |  |  |  |
| 25-34 | 29.8% | 25.0% | 0.95672 | 0.98514 | 21.5% | 18.5% | 16.0% |
| 35-44 | 23.0% | 21.6% | 0.98462 | 0.98514 | 18.6% | 16.0% | 13.8% |
| 45-54 | 21.8% | 23.2% | 1.01525 | 0.98514 | 19.9% | 17.2% | 14.8% |
| 55-64 | 21.1% | 18.9% | 0.97323 | 0.98514 | 16.3% | 14.0% | 12.1% |
| 65-74 | 12.9% | 13.5% | 1.00971 | 0.98514 | 11.6% | 10.0% | 8.6% |
| PI |  |  |  |  |  |  |  |
| 25-34 | 44.9% | 43.5% | 0.99220 | 0.96771 | 31.3% | 22.6% | 16.2% |
| 35-44 | 52.3% | 41.2% | 0.94203 | 0.96771 | 29.7% | 21.4% | 15.4% |
| 45-54 | 57.6% | 51.9% | 0.97399 | 0.96771 | 37.3% | 26.9% | 19.4% |
| 55-64 | 62.5% | 55.0% | 0.96848 | 0.96771 | 39.6% | 28.5% | 20.6% |
| 65-74 | 65.4% | 53.2% | 0.94985 | 0.96771 | 38.3% | 27.6% | 19.9% |
| Chol. |  |  |  |  |  |  |  |
| 25-34 | 4.74 | 4.80 | 1.00315 | 1.00021 | 4.81 | 4.82 | 4.83 |
| 35-44 | 5.24 | 5.23 | 0.99952 | 1.00021 | 5.24 | 5.25 | 5.26 |
| 45-54 | 5.44 | 5.45 | 1.00046 | 1.00021 | 5.46 | 5.47 | 5.48 |
| 55-64 | 5.30 | 5.29 | 0.99953 | 1.00021 | 5.30 | 5.31 | 5.32 |
| 65-74 | 5.15 | 5.05 | 0.99511 | 1.00021 | 5.06 | 5.07 | 5.08 |
| BMI |  |  |  |  |  |  |  |
| 25-34 | 26.7 | 27.2 | 1.00463 | 1.00497 | 28.6 | 30.0 | 31.5 |
| 35-44 | 27.4 | 27.8 | 1.00367 | 1.00497 | 29.2 | 30.7 | 32.3 |
| 45-54 | 27.9 | 28.1 | 1.00136 | 1.00497 | 29.5 | 31.0 | 32.6 |
| 55-64 | 28.1 | 28.8 | 1.00635 | 1.00497 | 30.2 | 31.8 | 33.4 |
| 65-74 | 27.6 | 29.3 | 1.01460 | 1.00497 | 30.8 | 32.3 | 34.0 |
| Diabetes |  |  | Absolute change per year |  |  |  |  |
| 25-34 | 0.7% | 0.0% | -0.00181 | 0.00083 | 0.8% | 1.7% | 2.5% |
| 35-44 | 1.9% | 2.8% | 0.00236 | 0.00083 | 3.7% | 4.5% | 5.3% |
| 45-54 | 4.7% | 4.6% | -0.00017 | 0.00083 | 5.4% | 6.2% | 7.1% |
| 55-64 | 9.6% | 11.2% | 0.00405 | 0.00083 | 12.0% | 12.8% | 13.7% |
| 65-74 | 14.6% | 14.8% | 0.00045 | 0.00083 | 15.6% | 16.4% | 17.2% |

**Table 4b. Women´s risk factor values by age group: Recent trends continue**

| **Age** | **2006** | **2010** | **Relative change per year** | **Weighted mean** | **2020** | **2030** | **2040** |
| --- | --- | --- | --- | --- | --- | --- | --- |
| SBP |  |  |  |  |  |  |  |
| 25-34 | 108.1 | 109.6 | 1.00350 | 0.99715 | 106.5 | 103.6 | 100.6 |
| 35-44 | 109.7 | 111.0 | 1.00290 | 0.99715 | 107.9 | 104.9 | 101.9 |
| 45-54 | 117.0 | 115.5 | 0.99680 | 0.99715 | 112.3 | 109.1 | 106.1 |
| 55-64 | 125.6 | 120.9 | 0.99045 | 0.99715 | 117.5 | 114.2 | 111.0 |
| 65-74 | 138.0 | 128.5 | 0.98247 | 0.99715 | 124.9 | 121.4 | 118.0 |
| Smoking |  |  |  |  |  |  |  |
| 25-34 | 24.2% | 23.9% | 0.99657 | 0.97097 | 17.8% | 13.2% | 9.9% |
| 35-44 | 24.2% | 16.2% | 0.90458 | 0.97097 | 12.1% | 9.0% | 6.7% |
| 45-54 | 22.5% | 21.1% | 0.98396 | 0.97097 | 15.7% | 11.7% | 8.7% |
| 55-64 | 19.1% | 20.2% | 1.01463 | 0.97097 | 15.0% | 11.2% | 8.3% |
| 65-74 | 16.4% | 13.6% | 0.95413 | 0.97097 | 10.1% | 7.5% | 5.6% |
| PI |  |  |  |  |  |  |  |
| 25-34 | 52.4% | 41.6% | 0.94411 | 0.95064 | 25.1% | 15.1% | 9.1% |
| 35-44 | 49.9% | 37.5% | 0.93100 | 0.95064 | 22.6% | 13.6% | 8.2% |
| 45-54 | 49.9% | 45.5% | 0.97713 | 0.95064 | 27.4% | 16.5% | 10.0% |
| 55-64 | 55.7% | 45.2% | 0.94895 | 0.95064 | 27.2% | 16.4% | 9.9% |
| 65-74 | 62.8% | 51.8% | 0.95291 | 0.95064 | 31.2% | 18.8% | 11.3% |
| Chol. |  |  |  |  |  |  |  |
| 25-34 | 4.43 | 4.54 | 1.00615 | 1.00247 | 4.65 | 4.77 | 4.89 |
| 35-44 | 4.87 | 4.83 | 0.99794 | 1.00247 | 4.95 | 5.07 | 5.20 |
| 45-54 | 5.30 | 5.49 | 1.00884 | 1.00247 | 5.63 | 5.77 | 5.91 |
| 55-64 | 5.80 | 5.76 | 0.99827 | 1.00247 | 5.90 | 6.05 | 6.20 |
| 65-74 | 5.83 | 5.76 | 0.99698 | 1.00247 | 5.90 | 6.05 | 6.20 |
| BMI |  |  |  |  |  |  |  |
| 25-34 | 25.3 | 25.7 | 1.00409 | 1.00287 | 26.4 | 27.2 | 28.0 |
| 35-44 | 26.2 | 26.7 | 1.00503 | 1.00287 | 27.5 | 28.3 | 29.1 |
| 45-54 | 27.0 | 27.1 | 1.00091 | 1.00287 | 27.9 | 28.7 | 29.5 |
| 55-64 | 27.5 | 27.3 | 0.99868 | 1.00287 | 28.1 | 28.9 | 29.8 |
| 65-74 | 27.6 | 28.3 | 1.00658 | 1.00287 | 29.1 | 30.0 | 30.9 |
| Diabetes |  |  | Absolute change per year |  |  |  |  |
| 25-34 | 0.5% | 1.0% | 0.00116 | 0.00164 | 2.6% | 4.3% | 5.9% |
| 35-44 | 1.0% | 1.3% | 0.00079 | 0.00164 | 3.0% | 4.6% | 6.3% |
| 45-54 | 2.1% | 2.8% | 0.00183 | 0.00164 | 4.4% | 6.1% | 7.7% |
| 55-64 | 4.2% | 4.1% | -0.00029 | 0.00164 | 5.7% | 7.3% | 9.0% |
| 65-74 | 7.4% | 10.3% | 0.00727 | 0.00164 | 12.0% | 13.6% | 15.3% |

**Table 5a. Men´s risk factor values by age group: Low risk scenario**

| **Age** | **2010** | **2020**  **35% low risk** | **2030**  **65% low risk** | **2040**  **100% low risk** |
| --- | --- | --- | --- | --- |
| SBP |  |  |  |  |
| 25-34 | 123.5 | **120.9** | **117.7** | 116.0 |
| 35-44 | 124.4 | **121.4** | **117.7** | 115.7 |
| 45-54 | 124.5 | **121.4** | **117.7** | 115.7 |
| 55-64 | 129.5 | **124.7** | **118.8** | 115.7 |
| 65-74 | 133.8 | **127.5** | **119.8** | 115.7 |
| Smoking |  |  |  |  |
| 25-34 | 25.0% | **16.3%** | **5.7%** | **0.0%** |
| 35-44 | 21.6% | **14.0%** | **4.9%** | **0.0%** |
| 45-54 | 23.2% | **15.0%** | **5.3%** | **0.0%** |
| 55-64 | 18.9% | **12.3%** | **4.3%** | **0.0%** |
| 65-74 | 13.5% | **8.7%** | **3.1%** | **0.0%** |
| PI |  |  |  |  |
| 25-34 | 43.5% | **28.3%** | **9.9%** | **0.0%** |
| 35-44 | 41.2% | **26.8%** | **9.4%** | **0.0%** |
| 45-54 | 51.9% | **33.7%** | **11.8%** | **0.0%** |
| 55-64 | 55.0% | **35.8%** | **12.5%** | **0.0%** |
| 65-74 | 53.2% | **34.6%** | **12.1%** | **0.0%** |
| Chol. |  |  |  |  |
| 25-34 | 4.80 | **4.62** | **4.40** | 4.28 |
| 35-44 | 5.23 | **4.99** | **4.70** | 4.54 |
| 45-54 | 5.45 | **5.13** | **4.75** | 4.54 |
| 55-64 | 5.29 | **5.03** | **4.71** | 4.54 |
| 65-74 | 5.05 | **4.87** | **4.66** | 4.54 |
| BMI |  |  |  |  |
| 25-34 | 27.2 | **26.3** | **25.2** | 24.6 |
| 35-44 | 27.8 | **27.0** | **26.0** | **25.5** |
| 45-54 | 28.1 | **27.2** | **26.1** | **25.5** |
| 55-64 | 28.8 | **27.6** | **26.2** | **25.5** |
| 65-74 | 29.3 | **28.0** | **26.4** | **25.5** |
| Diabetes |  |  |  |  |
| 25-34 | 0.0% | **0.0%** | **0.0%** | **0.0%** |
| 35-44 | 2.8% | **1.8%** | **0.6%** | **0.0%** |
| 45-54 | 4.6% | **3.0%** | **1.0%** | **0.0%** |
| 55-64 | 11.2% | **7.3%** | **2.5%** | **0.0%** |
| 65-74 | 14.8% | **9.6%** | **3.4%** | **0.0%** |

**Table 5b. Women´s risk factor values by age group: Low risk scenario**

| **Age** | **2010** | **2020**  **35% low risk** | **2030**  **65% low risk** | **2040**  **100% low risk** |
| --- | --- | --- | --- | --- |
| SBP |  |  |  |  |
| 25-34 | 109.6 | **109.8** | **109.9** | 110.0 |
| 35-44 | 111.0 | **110.7** | **110.2** | 110.0 |
| 45-54 | 115.5 | **115.2** | **114.9** | 114.7 |
| 55-64 | 120.9 | **118.7** | **116.1** | 114.7 |
| 65-74 | 128.5 | **123.7** | **117.8** | 114.7 |
| Smoking |  |  |  |  |
| 25-34 | 23.9% | **15.5%** | **5.4%** | **0.0%** |
| 35-44 | 16.2% | **10.5%** | **3.7%** | **0.0%** |
| 45-54 | 21.1% | **13.7%** | **4.8%** | **0.0%** |
| 55-64 | 20.2% | **13.1%** | **4.6%** | **0.0%** |
| 65-74 | 13.6% | **8.8%** | **3.1%** | **0.0%** |
| PI |  |  |  |  |
| 25-34 | 41.6% | **27.1%** | **9.5%** | **0.0%** |
| 35-44 | 37.5% | **24.4%** | **8.5%** | **0.0%** |
| 45-54 | 45.5% | **29.5%** | **10.3%** | **0.0%** |
| 55-64 | 45.2% | **29.4%** | **10.3%** | **0.0%** |
| 65-74 | 51.8% | **33.7%** | **11.8%** | **0.0%** |
| Chol. |  |  |  |  |
| 25-34 | 4.54 | **4.43** | **4.30** | 4.23 |
| 35-44 | 4.83 | **4.73** | **4.61** | 4.55 |
| 45-54 | 5.49 | **5.16** | **4.76** | 4.55 |
| 55-64 | 5.76 | **5.34** | **4.83** | 4.55 |
| 65-74 | 5.76 | **5.34** | **4.83** | 4.55 |
| BMI |  |  |  |  |
| 25-34 | 25.7 | **24.0** | **22.0** | 20.9 |
| 35-44 | 26.7 | **25.6** | **24.3** | **23.6** |
| 45-54 | 27.1 | **25.9** | **24.4** | **23.6** |
| 55-64 | 27.3 | **26.0** | **24.4** | **23.6** |
| 65-74 | 28.3 | **26.7** | **24.7** | **23.6** |
| Diabetes |  |  |  |  |
| 25-34 | 1.0% | **0.6%** | **0.2%** | **0.0%** |
| 35-44 | 1.3% | **0.9%** | **0.3%** | **0.0%** |
| 45-54 | 2.8% | **1.8%** | **0.6%** | **0.0%** |
| 55-64 | 4.1% | **2.6%** | **0.9%** | **0.0%** |
| 65-74 | 10.3% | **6.7%** | **2.4%** | **0.0%** |

**Table 6. Specific Beta Coefficients for Major Risk Factors:** **Data sources, values and comments**

Estimated β coefficients from multiple regression analyses for the relationship between absolute changes in population mean risk factors and % changes in coronary heart disease mortality for men and women, stratified by age.

| **Systolic Blood Pressure** | **Age groups (years)** | | | | |  |
| --- | --- | --- | --- | --- | --- | --- |
|  | 25-44 | 45-54 | 55-64 | 65-74 |  | |
| **Men** (hazard ratio per 20 mmHg) | **0.49** | **0.49** | **0.52** | **0.58** |  | |
| **Men** (log hazard ratio per 1 mmHg) | **-0.036** | **-0.035** | **-0.032** | **-0.027** |  | |
| Min | -0.029 | -0.028 | -0.026 | -0.022 |  | |
| Max | -0.043 | -0.042 | -0.039 | -0.032 |  | |
| **Women** (hazard ratio per 20 mmHg) | **0.40** | **0.40** | **0.49** | **0.52** |  | |
| **Women (log hazard ratio per 1 mmHg)** | **-0.046** | **-0.046** | **-0.035** | **-0.032** |  | |
| Min | -0.037 | -0.037 | -0.028 | -0.026 |  | |
| Max | -0.055 | -0.055 | -0.042 | -0.039 |  | |
| Source: Prospective studies collaborative meta-analysis, Lancet 2002[^13^](#_ENREF_13)  *UNITS: % mortality change per 20 mmHg change in Systolic BP | | | | | |  |

| **Cholesterol** | **Age groups (years)** | | | | | | | | |  | |
| --- | --- | --- | --- | --- | --- | --- | --- | --- | --- | --- | --- |
|  | 25-44 | 45-54 | | 55-64 | | | 65-74 | |  | | |
| **Mortality reduction per 1 mmol/l**  Men | **0.55** | **0.53** | | **0.36** | | | **0.21** | |  | | |
| Women | **0.57** | **0.52** | | **0.35** | | | **0.23** | |  | | |
| **Log coefficient**  Men | -0.799 | -0.755 | | -0.446 | | | -0.236 | |  | | |
| Women | -0.844 | -0.734 | | -0.431 | | | -0.261 | |  | | |
| Source: Prospective studies collaborative meta-analysis, Lancet 2007.[^16^](#_ENREF_16)  Body Mass Index (BMI) | **Age groups (years)** | | | | | | | | |  | |
|  | <44 | | 45-59 | | 60-69 | 70-79 | |  | | |  |
| Risk reduction per 1 kg/m^2^:  James Asia Pacific data | 0.1100 | | 0.0900 | | 0.0500 | 0.0400 | |  | | |  |
| Asia Pacific age gradient therefore: | 1.22 | | **1.00** | | 0.56 | 0.44 | |  | | |  |
| Bogers relative risks,  CHD deaths per 5 kg/m^2^ |  | | **1.16** | |  |  | |  | | |  |
| Age specific relative risks per 1 kg/m^2^,  Applying age gradients from James et al | 1.04 | | **1.03** | | 1.02 | 1.01 | |  | | |  |
| **Men & Women, log coefficients*** | **0.0363** | | **0.0297** | | **0.0165** | **0.0132** | |  | | |  |
| Minimum values | 0.0255 | | 0.0209 | | 0.0116 | 0.0093 | |  | | |  |
| Maximum values *(from James et al)* | 0.0466 | | 0.0381 | | 0.0212 | 0.0169 | |  | | |  |
| Source: Bogers et al. 2006,[^17^](#_ENREF_17) James et al. 2004.[^18^](#_ENREF_18) *UNITS: % mortality change per 1 kg/m^2^ change in BMI  Strengths: Large number of studies included. Adjusted for blood pressure, total cholesterol, and physical activity. 95% CIs also provided. Limitations: Observational data; age gradient applied from James study. | | | | | | | | | |  | |

**Table 7. Relative Risks Used in the Icelandic IMPACT Model for Smoking, Diabetes and Physical Inactivity for Coronary Heart Disease Mortality.** (Best, Minimum and Maximum Estimates from the InterHeart Study**^a^**)

|  | **Both sexes** | | **Men** | | **Women** | |
| --- | --- | --- | --- | --- | --- | --- |
|  | **Young** | **Old** |  | **>55 years** |  | **> 65 years** |
| Lifestyle factors |  |  |  |  |  |  |
| **Smoking** | **3.33 (2.86-3.87)** | **2.44 (2.10-2.84)** | **3.33 (2.80-3.95)** | **2.52 (2.15-2.96)** | **4.49 (3.11-6.47)** | **2.14 (1.35-3.39)** |
| **Fruit and vegetables** | 0.69 (0.58-0.81) | 0.72 (0.6-0.85) | 0.72 (0.59-0.88) | 0.77 (0.64-0.93) | 0.62 (0.44-0.87) | 0.55 (0.38-0.80) |
| **Exercise** | **0.95 (0.79-1.14)** | **0.79 (0.66-0.94)** | **1.02 (0.83-1.25)^b^** | **0.79 (0.66-0.96)** | **0.74 (0.49-1.10)** | **0.75 (0.46-1.22)** |
| **Alcohol** | 1.00 (0.85-1.17) | 0.85 (0.73-1.00) | 1.03 (0.87-1.23) | 0.86 (0.73-1.01) | 0.74 (0.41-1.31) | 0.83 (0.49-1.42) |
| **Hypertension** | 2.24 (1.93-2.60) | 1.72 (1.52-1.95) | 1.99 (1.66-2.39) | 1.72 (1.49-1.98) | 2.94 (2.25-3.85) | 1.82 (1.39-2.38) |
| **Diabetes** | **2.96 (2.40-3.64)** | **2.05 (1.71-2.45)** | **2.66 (2.04-3.46)** | **1.93 (1.58-2.37)** | **3.53 (2.49-5.01)** | **2.59 (1.78-3.78)** |
| **Abdominal obesity** | 1.79 (1.52-2.09) | 1.50 (1.29-1.74) | 1.83 (1.52-2.20) | 1.54 (1.30-1.83) | 1.58 (1.14-2.20) | 1.22 (0.88-1.70) |
| **Psychosocial** | 2.87 (2.19-3.77) | 2.43 (1.86-3.18) | 2.62 (1.91-3.60) | 2.45 (1.82-3.29) | 3.92 (2.26-6.79) | 2.31 (1.22-4.39) |
| **High ApoB/ApoA1 ratio** | 4.35 (3.49-5.42) | 2.50* (2.05-3.05) | 4.16 (3.19-5.42) | 2.51 (2.00-3.15) | 4.83 (3.19-7.32) | 2.48 (1.60-3.83) |

(and see Introduction for a worked example)

**Yusuf, InterHeart Study. Lancet 2004.^[14](#_ENREF_14)^ *Odds ratios for relative effect of risk factors*** *(99% Confidence Intervals, NOT 95%)*

Smoking, adverse lipid profile, hypertension, and diabetes had a greater relative effect on risk of acute myocardial infarction in younger than older individuals

**^a^**Global InterHeart values were used in the Icelandic IMPACT Model

^b^The InterHeart study quoted a value of only 1.02 for exercise in men aged <55 years. This was clearly an outlier. We have therefore assumed a value of 0.77 in line with men and women in the other age groups, and consistent with most other studies.

gg g

# Table 8. Iceland Impact Model Risk Factor Methodology: Rationale for choice of regression or PARF approaches for specific risk factors

This table explains the rationale for choosing the best approach for each risk factor: regression based on absolute change in the risk factor*, regression based on relative change in the risk factor*, or population attributable risk fraction (PARF).

We also specify the best data source for each.

*Absolute and Relative beta regression approaches are illustrated earlier in the Supplementary Appendix.

An absolute beta regression coefficient quantifies the CHD mortality reduction for each UNIT change in risk factor, e.g. mmHg change for BP, or mg/dl change for cholesterol

A RELATIVE beta regression coefficient quantifies the CHD mortality reduction for each % relative change in risk factor, e.g. a 12 mmHg fall in SBP, from 120 mmHg to 108 mmHg, would represent a **10%** relative decrease (12/120).

| **Risk Factor** | **Source** | Strengths | **Limitations** | **Comments and recommend-**  **ation** | **DPP value in Icelandic Model** (contribution to total CHD mortality fall) |
| --- | --- | --- | --- | --- | --- |
| **Blood pressure** |  |  |  |  |  |
| 1. Systolic BP: regression using absolute beta approach | PSC 2002^13^ | Large meta-analyses include Swedish and European data. Age and sex stratified.  SBP preferable to  DBP, because stronger relationship with CHD deaths. | Observational data- assume complete reversibly of risk. | CURRENT APPROACH  Supersedes relative approach. | 72 (24.3%) |
| 2. PARF | Midspan | Original approach in Scottish IMPACT Model. | Sensitive to reference value and category cut-offs. Estimated DPPs always appeared very low. | Obsolete | - |

| **Cholesterol** |  |  |  |  |  |
| --- | --- | --- | --- | --- | --- |
| 1. Regression using absolute Beta | Law et al, meta-analysis | Large meta-analysis, split by age and sex; cohort and RCT results very consistent; supported by more recent reviews | Published in 1994 | CURRENT APPROACH | 95 (32.3%) |
| 2. PARF using quintiles | Midspan | Used in 1996 | Sensitive to reference value and category cut-offs. | Obsolete since 1997 | - |
| **BMI** |  |  |  |  |  |
| 1.Regression using absolute Beta | Bogers et al 2006^[17](#_ENREF_17)^ | Large meta-analysis with US data, Broadly consistent with Asian and PSC analyses; age-splits taken from James et al. Adjusted for major confounders: smoking, cholesterol, blood pressure, and physical activity | An “upstream” CHD risk factor. CHD risk partly or wholly mediated through “downstream factors: BP, cholesterol and impaired glucose tolerance. DPP values consistent with earlier US studies. | CURRENT APPROACH  Potential confounding addressed by using this adjusted value | -13 (-4.4%) |
| 2. PARF using OBESITY quintiles | Inter-  Heart[^19^](#_ENREF_19) | Large, global study including data from Sweden | Sensitive to reference value and category cut-offs. Under-estimation likely. | An arbitrary approach to a continuous variable.  Superseded | -22  (-7.4%) |

| **Smoking** |  |  |  |  |  |
| --- | --- | --- | --- | --- | --- |
| 1. PARF | Inter-  Heart[^14^](#_ENREF_14) | Log linear.  InterHeart large, global study including Swedish data.  RRs consistent with other studies. Appropriate for a dichotomous variable. | Regression approach might provide useful alternative approach? | CURRENT APPROACH | 65 (22.0%) |
| 2. Regression using absolute beta | Vartiainen 1994[^20^](#_ENREF_20) | Used in earlier IMPACT Models. Result consistent with PARF approach. | Not dichotomous. Not log-linear | Superseded | 148 (50.0%) |

| **Diabetes** |  |  |  |  |  | |  |  |  |
| --- | --- | --- | --- | --- | --- | --- | --- | --- | --- |
| 1. PARF approach | Inter  Heart^14^ | Large, global study including Swedish data.  RRs consistent with other studies. Appropriate method for dichotomous variable. | Case control study, albeit huge. | CURRENT APPROACH | -14 (-4.6%) | |  |  |  |
| 2. Regression approach | - | - | Appropriate Betas not identified, and methodologically dubious | Not attempted | - | |  |  |  |
|  |  |  |  |  |  |  | |  |  |
| **Physical activity** |  |  |  |  |  | |  |  |  |
| 1. PARF approach | Inter-  Heart^14^ | Large, global study including US data.  RRs consistent with other studies. Appropriate method for dichotomous variable. | Alternative PARF methods possible. Important to use independent RR values. (Aim to examine activity sub-categories in future studies) | CURRENT APPROACH | 16 (5.4%) | |  |  |  |
| 2. Regression approach | - | - | Appropriate Betas do not exist, and methodologically dubious | Not attempted | - | |  |  |  |
|  |  |  |  |  |  | |  |  |  |

**REFERENCES**

**Figure 1.** Risk factor development in the past and three future risk factor scenarios.


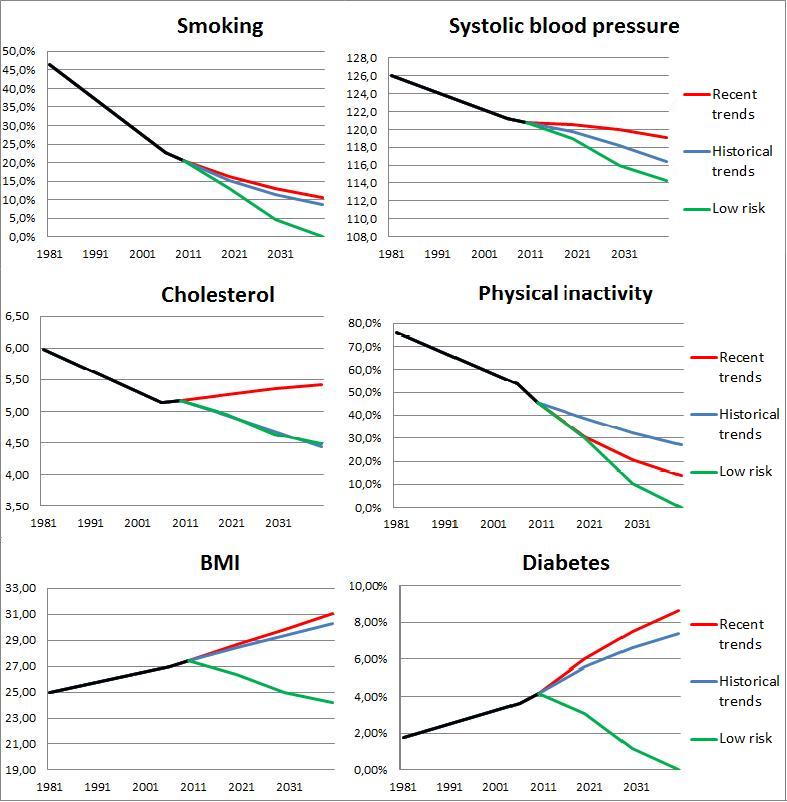


1. Capewell S, Beaglehole R, Seddon M, McMurray J. Explanation for the decline in coronary heart disease mortality rates in Auckland, New Zealand, between 1982 and 1993. Circulation 2000;102:1511-6.

2. Capewell S, Morrison CE, McMurray JJ. Contribution of modern cardiovascular treatment and risk factor changes to the decline in coronary heart disease mortality in Scotland between 1975 and 1994. Heart (British Cardiac Society) 1999;81:380-6.

3. Laatikainen T, Critchley J, Vartiainen E, Salomaa V, Ketonen M, Capewell S. Explaining the decline in coronary heart disease mortality in Finland between 1982 and 1997. American journal of epidemiology 2005;162:764-73.

4. Unal B, Critchley JA, Capewell S. Explaining the decline in coronary heart disease mortality in England and Wales between 1981 and 2000. Circulation 2004;109:1101-7.

5. Unal B, Critchley JA, Capewell S. Modelling the decline in coronary heart disease deaths in England and Wales, 1981-2000: comparing contributions from primary prevention and secondary prevention. BMJ (Clinical research ed) 2005;331:614.

6. Critchley J, Liu J, Zhao D, Wei W, Capewell S. Explaining the increase in coronary heart disease mortality in Beijing between 1984 and 1999. Circulation 2004;110:1236-44.

7. Ford ES, Ajani UA, Croft JB, et al. Explaining the decrease in U.S. deaths from coronary disease, 1980-2000. The New England journal of medicine 2007;356:2388-98.

8. Bjorck L, Rosengren A, Bennett K, Lappas G, Capewell S. Modelling the decreasing coronary heart disease mortality in Sweden between 1986 and 2002. European heart journal 2009;30:1046-56.

9. Aspelund T, Gudnason V, Magnusdottir BT, et al. Analysing the large decline in coronary heart disease mortality in the Icelandic population aged 25-74 between the years 1981 and 2006. PloS one 2010;5:e13957.

10. Unal B, Critchley J, Capewell S. IMPACT, a validated, comprehensive coronary heart disease model. In. Liverpool, United kingdom: University of Liverpool 2006. (Accessed April 30, at <http://www.liv.ac.uk/PublicHealth/sc/bua/impact.html)>.

11. Stamler J, Stamler R, Neaton JD, et al. Low risk-factor profile and long-term cardiovascular and noncardiovascular mortality and life expectancy: findings for 5 large cohorts of young adult and middle-aged men and women. JAMA : the journal of the American Medical Association 1999;282:2012-8.

12. Daviglus ML, Stamler J, Pirzada A, et al. Favorable cardiovascular risk profile in young women and long-term risk of cardiovascular and all-cause mortality. JAMA : the journal of the American Medical Association 2004;292:1588-92.

13. Lewington S, Clarke R, Qizilbash N, Peto R, Collins R. Age-specific relevance of usual blood pressure to vascular mortality: a meta-analysis of individual data for one million adults in 61 prospective studies. Lancet 2002;360:1903-13.

14. Yusuf S, Hawken S, Ounpuu S, et al. Effect of potentially modifiable risk factors associated with myocardial infarction in 52 countries (the INTERHEART study): case-control study. Lancet 2004;364:937-52.

15. Briggs A, Sculpher M, Buxton M. Uncertainty in the economic evaluation of health care technologies: the role of sensitivity analysis. Health economics 1994;3:95-104.

16. Lewington S, Whitlock G, Clarke R, et al. Blood cholesterol and vascular mortality by age, sex, and blood pressure: a meta-analysis of individual data from 61 prospective studies with 55,000 vascular deaths. Lancet 2007;370:1829-39.

17. Bogers RPHR, Boshuizen H, Woodward M, et al. Overweight and obesity increase the risk of coronary heart disease: a pooled analysis of 30 prospective studies. European Journal of Epidemiology 2006;21((supplement):107.).

18. James WPTJ-LR, Mhurchu CN, et.al. Overweight and obesity (high body mass index). Comparative quantification of risk. Global and regional burden of disease attributable to selected major risk factors. In: World Health Organization; 2004.

19. Yusuf S, Hawken S, Ounpuu S, et al. Obesity and the risk of myocardial infarction in 27,000 participants from 52 countries: a case-control study. Lancet 2005;366:1640-9.

20. Vartiainen E, Puska P, Pekkanen J, Tuomilehto J, Jousilahti P. Changes in risk factors explain changes in mortality from ischaemic heart disease in Finland. BMJ (Clinical research ed) 1994;309:23-7.
